# The state of mechanistic research in the evidence‐based medicine era: A sandwalk between triangulation and hierarchies

**DOI:** 10.1113/EP092157

**Published:** 2025-02-21

**Authors:** Ronan M. G. Berg, Cody G. Durrer, Jan Kyrre Berg Olsen Friis, Mathias Ried‐Larsen

**Affiliations:** ^1^ Centre for Physical Activity Research Copenhagen University Hospital–Rigshospitalet Copenhagen Denmark; ^2^ Department of Clinical Physiology and Nuclear Medicine Copenhagen University Hospital–Rigshospitalet Copenhagen Denmark; ^3^ Department of Clinical Medicine, Faculty of Health and Medical Sciences University of Copenhagen Copenhagen Denmark; ^4^ Neurovascular Research Laboratory, Faculty of Life Sciences and Education University of South Wales Pontypridd UK; ^5^ Section of Health Services Research, Department of Public Health, Faculty of Health and Medical Sciences University of Copenhagen Copenhagen Denmark

Formally, to have evidence is to have a ‘conceptual warrant for belief or action’ (Goldenberg, 2006), and to paraphrase Frank Herbert (1926–1980) in his novel *Dune*, evidence is certainly the melange, or ‘spice,’ around which all science is centred (Herbert, 1965). The concept of evidence that seems to dominate most biomedical sciences these days is that advocated by the evidence‐based medicine (EBM) movement, which emerged as a new paradigm in the early 1990s with the ambition of basing clinical practice and teaching strictly on evidence as defined within the ‘hierarchy of evidence’ (Timmermans & Mauck, 2005) (Figure 1). Despite our enduring quest for truth within the field of experimental physiology, which seems more important than ever these days (Drummond & Tipton, 2024), mechanistic studies are consistently placed at the bottom in various incarnations of this hierarchy (Djulbegovic & Guyatt, 2017; Williamson, 2019). Does this mean that all our efforts as researchers within experimental physiology and other lines of mechanistic research contribute nothing more than ‘low quality’ evidence? Certainly not! Here, we will make the case that while EBM and experimental physiology benefit from each other and are complementary in many ways, they operate with fundamentally different frameworks. The main scientific philosophical concepts we will discuss are summarised in Box 1. We will make the case that EBM‐based criteria for what constitutes ‘good evidence’ cannot uncritically be extrapolated to mechanistic research. And vice versa for that matter.

**FIGURE 1:**
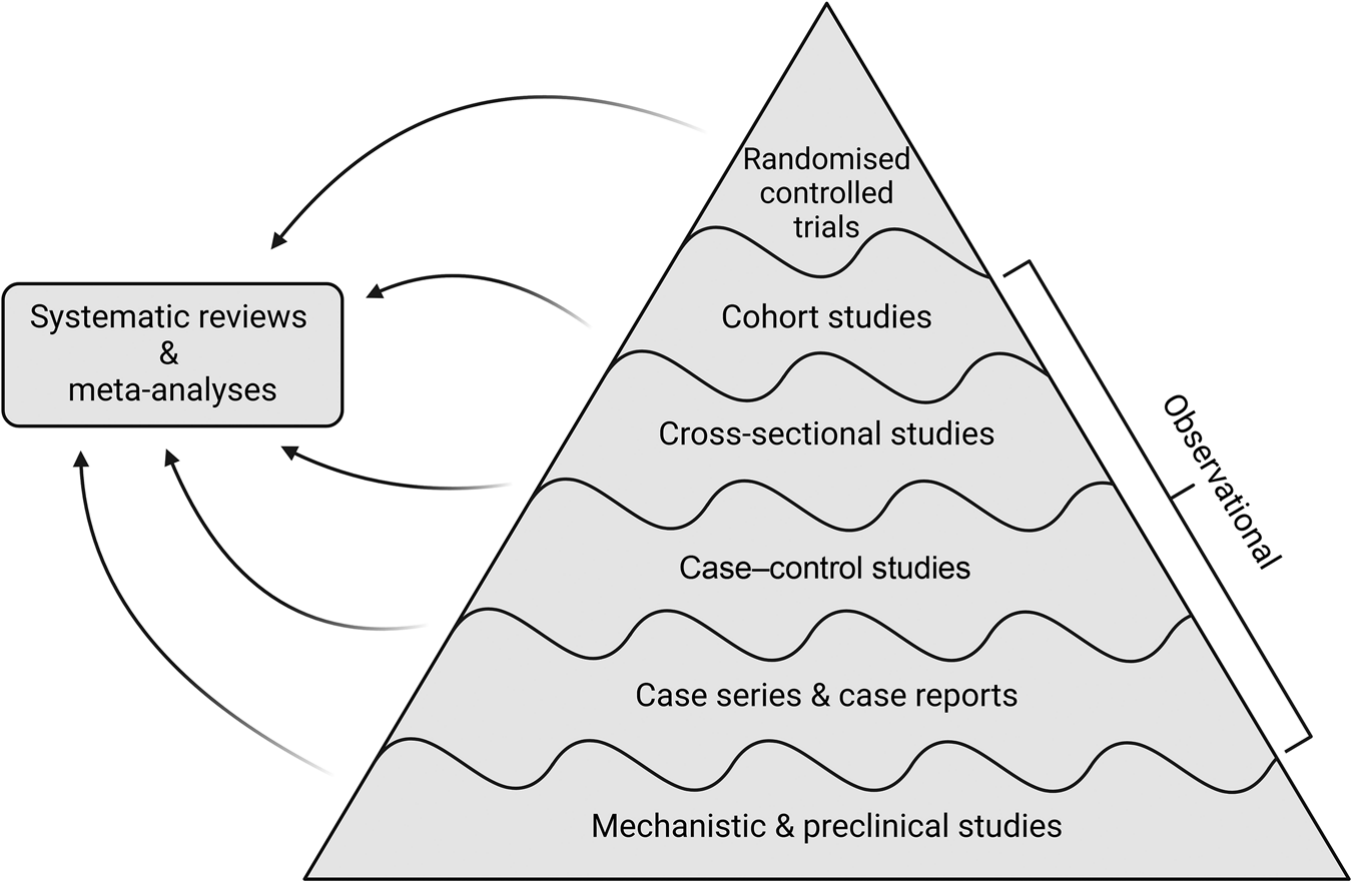
Example of the ‘evidence hierarchy’ in evidence‐based medicine. Well‐conducted randomised controlled trials sit at the top of the pyramid, as this is the primary design through which causal evidence is generated. As the reader moves to designs lower on the pyramid, the ability to draw purely causal conclusions disappears and evidence‐supporting causality in an EBM framework lessens. The lines separating the different study designs are depicted as waves to indicate that the evidence generated by a high quality ‘lower level’ study design can be more valid than that generated by a low quality ‘higher level’ study design. In an EBM framework, mechanistic human studies would appear at the bottom of the pyramid due to their lack of validity to directly inform clinical decision‐making. Systematic reviews and meta‐analyses, despite often appearing at the top of evidence pyramids, are instead depicted beside to indicate their strength being reliant upon the underlying research they synthesise from. Created with BioRender.com.

BOX 1 **Key philosophical concepts**

*Empiricism*: The idea that knowledge comes from sensory experience and observation. It emphasises that all knowledge must be grounded in empirical evidence, meaning it must be rooted in observable phenomena. Empiricism rejects the notion that innate ideas or purely logical deduction can provide meaningful knowledge of the world.

*Instrumentalism*: A view often linked to pragmatism; it holds that theories are valuable only insofar as they are useful in solving problems. Theories are seen as tools, not necessarily reflective of the true nature of the world, but as instruments for achieving practical outcomes.

*Positivism*: An extension of empiricism, this philosophy, which historically exists in several incarnations, asserts that knowledge of the world should be based strictly on quantifiable data from observation and experimentation. It maintains that empirical data are the only valid sources of knowledge, rejecting metaphysical explanations or speculation as unscientific. Positivism is closely related to scientific materialism, as both reject non‐material explanations and emphasise empirical investigation, but positivism focuses specifically on observable and measurable phenomena.

*Pragmatism*: A practical approach that emphasises knowledge as a tool for solving real‐world problems, particularly in settings like clinical medicine. Pragmatism values the effectiveness of interventions over theoretical completeness or certainty, focusing on what works in practice, even if the underlying theory is uncertain or incomplete.

*Scientific realism*: The view that a real world, including both observable and unobservable entities, exists independently of our perceptions or theories. Scientific realism holds that scientific theories aim to describe and explain the true nature of this world, and when a theory is successful, it is likely because it accurately reflects reality. Scientific realism aligns with scientific materialism in its commitment to understanding the world as independent of human perception, though it also accepts that unobservable entities, such as subatomic particles, are real and can be studied through scientific inquiry.

*Scientific materialism*: A philosophy closely aligned with positivism and often linked to scientific realism. It holds that all phenomena, including mental processes and consciousness, can be fully explained by physical matter and its interactions. Like positivism, scientific materialism emphasises empirical investigation as the primary means of understanding the world, rejecting non‐material explanations as unscientific. However, while positivism focuses strictly on observable and quantifiable data, scientific materialism extends this to assert that even unobservable processes, such as those studied in physics and biology, must ultimately be rooted in material causes. This places it within the broader framework of scientific realism, as both posit that a real world exists independently of human perception and that it can be known through science.

The field of experimental physiology emerged in France and Germany during the early 19th century, liberating physiology from natural philosophy, which had dominated its early history (Bailey et al., 2023a; Cunningham, 2002). Consequently, scientists in this new field radically discarded the concept of vitalism, which posited a mystical life force as the distinguishing element between living and non‐living matter. Instead, life's processes were to be determined within the realms of scientific materialism, that is, by the laws of physics and chemistry alone and thus requiring analysis through these exact scientific disciplines (Coleman, 1987; Culotta, 1970). While this new field of experimental physiology was a basic science, its scope was quite practical: to inform clinical practice by providing physicians with a theoretical framework upon which to base their decisions (Goldenberg, 2006).

At its core, experimental physiology is a positivistic science, founded in so‐called scientific realism, which is based on the premise that a real world exists, with its own structural and functional properties, which can be objectively studied through experimentation (Goldenberg, 2006). Although it underwent gradual modification, this positivism, with its reliance on deductive and inductive reasoning from mechanistic principles and theories, dominated clinical medicine for most of the 20th century (Timmermans & Mauck, 2005). EBM specifically evolved as a reaction against this, as mechanistic studies and theories were repeatedly found to be ineffective in predicting treatment effects in the clinical setting, and sometimes even leading to practices that turned out to be directly harmful. Even from the standpoint of scientific realism, this makes sense, because experimental models and conditions rarely reflect clinical reality in all its complexity, such that seeking for monocausal relationships for phenomena that are in reality multicausal has a high likelihood of failure. Furthermore, when one mechanism is targeted, its operation can be stopped or screened off by another causal factor, because several mechanisms typically operate simultaneously in vivo. Only the joint knowledge of all mechanisms and their interactions would suffice for taking mechanistic causal claims as a basis for decisions regarding interventions, something that even the most skilled experimentalist would find extremely challenging, if possible at all!

EBM advocated for a radically different approach. Rather than relying on mechanistic theories, treatment effects should be evaluated specifically on the basis of clinical and patient‐centred outcomes. These include mortality, hospital discharge or readmission, changes in treatments and health‐related quality of life, as well as through the systematic registration of adverse events (Timmermans & Mauck, 2005) and/or by changes in context‐specific biomarkers that are known to correlate with a given clinical outcome of interest (Manyara et al., 2024). While the exact philosophical underpinnings of EBM are unclear and a matter of debate (Djulbegovic & Guyatt, 2017; Kulkarni, 2005; Thomas, 2023), it is undeniably positivistic, yet clearly opposes the scientific realism of mechanistic reasoning, at least in the context of clinical decision‐making. Rather, EBM has clear elements of pragmatism, that is, emphasising the practical application of knowledge over theoretical reasoning, such that knowledge comes from observations and experiences rather than innate ideas or reason. Taken to the extreme, this implies that it is only relevant if a given cause–effect relationship is present or not, and any theoretical considerations of how and why are irrelevant (Goldenberg, 2006). While the purpose is thus not theory‐building with the goal of understanding the real world, EBM mainly has an instrumentalistic approach to theories. This implies that EBM may accept theory‐building as a tool for predicting and controlling phenomena, but without considering such theories true descriptions of the real world. This is consistent with the fact that studies conducted within an EBM framework often test hypotheses regarding treatment effects based on mechanistic theories. Indeed, it would rarely be considered ethical to conduct a clinical trial on a new treatment with no theoretical rationale to support its benefit.

When it comes to obtaining evidence to inform clinical decision‐making, EBM requires that the efficacy and adverse events of a treatment are systematically evaluated on patients in the clinical setting. The primary focus is on design to minimise confounding variables; this is principally achieved through randomised controlled trials (Williamson, 2019). However, it is important to note that considerations regarding what constitutes ‘good evidence’, as depicted in the hierarchy of evidence, specifically relate to the ability to inform clinical decision‐making. Thus, the placement of randomised trials at the top and mechanistic studies at the bottom does not reflect a difference between their implicit scientific value, but merely in their utility for directly informing clinical decision‐making and health recommendations. Hence, just as mechanistic studies are insufficient for informing clinical decision‐making, clinical studies—here understood as studies on patient populations focusing on clinical and patient‐centred outcomes—are rarely suited for making causal claims regarding mechanisms (Maziarz, 2023).

Here, it may be worth noting that due to their different philosophical underpinnings, EBM and mechanistic research operate with different concepts of causality. As EBM has its philosophical roots in pragmatism, it builds on a strictly manipulative causality concept, asserting that the experimental manipulation of a cause will result in the manipulation of an effect, thus practically making randomised controlled trials the only means for estimating the average treatment effect and potential harms, provided that they are well designed and well conducted. As such, the best evidence is obtained when the same hypothesis regarding a treatment effect repeatedly resists falsification in similarly conducted studies in similar populations.

In contrast, causality is best appreciated as pluralistic, relying on both manipulative and descriptive reasoning in mechanistic research (Maziarz, 2023; Williamson, 2019). While causal claims can strictly be applied only to the specific experimental setting, model and system under study in mechanistic research, scientific realism permits interpretation of cause–effect relationships within its own adaptable theoretical framework, provided they can be replicated and consistently resist experimental falsification. This collective evidence is then used to make general claims about physiological mechanisms. Indeed, it is this ‘inductive leap’ of scientific realism that builds theories from which new hypotheses can be formulated, including clinical studies conducted within an EBM framework, such that these do not rely solely on incidental discoveries to generate new ideas for potential therapies.

While the use of mechanistic studies to inform working hypotheses for clinical trials is the modus operandi, it works both ways. Clinical trial results can also yield incidental findings that inspire new mechanistic hypotheses, prompting further research. A notable example is the Women's Health Initiative trial, which unexpectedly revealed that combined oestrogen–progestin hormone replacement therapy increased the risk of breast cancer and cardiovascular disease compared to placebo in post‐menopausal women (Rossouw et al., 2002). This was contrary to the prevailing belief that hormone replacement therapy might be protective against these conditions and given that the Women's Health Initiative trial did not provide the actual mechanisms, the findings led to a long line of mechanistic studies into the roles of oestrogen and progestins in both carcinogenesis and vascular function. Similarly, a randomised controlled trial of intensive insulin treatment to maintain strict blood glucose control in critically ill surgical patients showed that this reduced mortality specifically due to septic complications (Van den Berghe et al., 2001), which led to subsequent mechanistic studies on the immune‐modulatory effects of insulin and related peptides.

Mechanistic research has learned many important lessons from EBM, particularly the emphasis on design, where random assignment to treatment and control groups ensures, at least in principle, an equal distribution of unknown confounders, although these may be unequally distributed by chance, particularly if the sample size is small. This is notably important in clinical trials because the risk of unknown confounders that are unbalanced between groups is particularly high for clinical populations. Experimental physiology and related fields within mechanistic research, such as pharmacology, biochemistry and microbiology, encompass a wide range of research methods in both laboratory and applied (i.e., environmental or clinical) settings. For experimental physiology, this includes in vitro, ex vivo and in vivo models, which are applied to a variety of systems, including individual cells, cells in culture, tissue preparations and various isolated organ preparations, as well as animals and studies involving human subjects. From its inception, experimental physiology drew from the controlled experiments that formed the basis of physics and chemistry (Coleman, 1987). In its simplest form, the controlled experiment involves predicting an event by assessing the impact of changes in preconditions within a highly controlled environment. The experimental conditions are then systematically modified and adapted to manipulate or observe spontaneous changes in an independent variable while standardising conditions to rule out the effects of other confounding variables. This is done as part of an iterative process that often involves multiple cycles of switching between deduction and induction, thereby identifying cause–effect relationships between the independent and dependent variables. While successful randomisation is in principle the only means of eliminating both known and unknown confounders, several other procedures may be used to effectively minimise this in mechanistic studies. This includes various experimental manipulations that target the biological pathways under study through pharmacological, environmental, behavioural and/or genetic activation or inhibition. This may be relevant when randomisation is either impossible or unethical, such as when the natural history of a disease is studied or when a disease is compared to the healthy state, or when the exposure under study is assumed to have harmful effects, as based on theoretical reasoning or other lines of empirical evidence. Furthermore, controlling for various known confounders in the statistical analysis can also be effective here, for example, via inclusion of covariates in the analysis or by weighted regression.

Despite some thematic overlap, it is important to note that although experimental physiology has historically been conceived to inform clinical medicine, it is a basic science with various aspects of physiology having a much broader scope than health‐related outcomes. Challenges may thus arise when mechanistic studies in humans are classified as clinical studies for legal or ethical reasons, often leading to the mistaken belief that this classification also applies to the scientific aspects of the study (Richter et al., 2024). Clearly, when conducting studies on humans in various applied (including the clinical) settings, the risk of confounders is higher than in the controlled laboratory setting—for we can only to a very limited extent control previous or concurrent factors, including various genetic and environmental exposures, that may contribute to the observed cause–effect relationships. Of note, the same applies to studies on non‐laboratory animals, such as pets, livestock and wild animals.

While randomisation is probably the most powerful tool for avoiding unbalanced unknown confounders between groups, it is important to note that randomisation in itself does not control for confounders if improperly implemented or if sample sizes are too small. Along with imprecise outcome assessments, and poorly described experimental set‐ups, this increases the risk of so‐called magnitude and sign errors (Gelman & Carlin, 2014). Furthermore, properly performed randomisation with adequate sample sizes is not always possible or even relevant in mechanistic studies, particularly in the applied setting. In any event, it is useful to clearly distinguish actual hypothesis‐testing studies from exploratory studies. We posit that many different designs can be used to test mechanistic hypotheses in applied settings, but the highest degree of certainty is achieved when based on a randomised controlled design, in which it is possible to prioritise outcomes and define an expected effect size, that is, a physiologically relevant difference, for sample size calculation. Consequently, most studies in the applied setting are exploratory, as they rarely focus on a single variable or outcome but rather on several interconnected variables that together show a pattern of change, rendering the prioritisation of outcomes meaningless. Furthermore, it is often impossible to define the minimal difference that is mechanistically relevant. However, as we will argue below, results from exploratory studies are by no means to be considered ‘low quality evidence’ as in EBM.

How to define a minimal physiological difference that is mechanistically relevant is a matter of considerable debate, with some emphasising that a physiologically relevant difference should be determined by either theoretical or statistical predictions or field‐established thresholds (Mesquida & Lakens, 2024; Williams et al., 2023), and others arguing that it should simply be set at the established minimally clinically relevant difference (Ciani et al., 2023). However, the latter is rarely available for the given context and furthermore only reflects the utility of the variable as a surrogate measure of a given clinical outcome, and thus not its mechanistic involvement. Alternatively, one could argue that any measurable change may in principle be physiologically relevant, thus highlighting the importance of the validity and reliability of the specific physiological measurement technique at play (Hartmann et al., 2023). However, unless one has unlimited resources, it often becomes impossible to falsify a hypothesis based on this, making relatively low statistical power a prerequisite in many physiological studies (Berg et al., 2024).

A major strength of EBM lies in its ability to incorporate systematic reviews and meta‐analyses, which synthesise findings from multiple studies and provide an assessment of the degree of the certainty of existing evidence regarding treatment effects on well‐defined outcomes in clinical populations. While mechanistic evidence draws on collective evidence from different lines of research rather than individual studies, it can still benefit from a similar approach to data synthesis through systematic reviews and meta‐analyses to assess the degree of certainty of evidence in favour of a given mechanism. Rather than focusing on randomisation as embedded within the evidence hierarchy, this should be based on so‐called triangulation (Box 2). Here, triangulation involves the use of multiple models, systems and settings to address one question, each with its own unrelated assumptions, strengths and weaknesses, because results that agree across these different models and systems are less likely to be artefacts (Munafò & Smith, 2018). This approach would also ensure that findings from many mechanistic studies, both hypothesis‐testing and exploratory, including the many individual studies that are underpowered due to constraints of scale, resources and ethical considerations (Berg et al., 2024; Schulz & Grimes, 2005), all contribute meaningfully to the collective evidence base.

BOX 2 **Triangulation principles in mechanistic research**

- The different approaches address the same underlying question.
- The key sources of bias for each approach are explicitly acknowledged.
- For each approach, the expected directions of all key sources of potential bias are made explicit, where feasible.
- Ideally, some of the approaches being compared will have potential biases that are in opposite directions.
- Ideally, results from more than two approaches — which have different and unrelated key sources of potential biases — are compared.

Based on (Munafò & Smith, 2018).

It is clear that both EBM and mechanistic research strive to attain evidence in the form of a conceptual warrant for belief (mechanistic research) and action (EBM), respectively, but from quite different philosophical standpoints. However, as the global replication crisis within all life science research has intensified since the turn of the millennium (Bailey et al., 2023b; Schooler, 2014), it is relevant to discuss and establish what constitutes ‘good evidence’ in experimental physiology and other types of mechanistic research. This should be done while keeping in mind that succumbing entirely to the principles of EBM would devalue more than 150 years of research within our field; simply put, it is egregiously bad practice to judge what is good or bad evidence based on incorrect premises! However, just as our field has benefited from the preregistration of study protocols and analysis plans to avoid selective analysis and publication bias (Christensen et al., 2025), an approach also endorsed by the Registered Report publication type in this journal (Rasmussen et al., 2025), may it be time to develop field‐specific consensus guidelines for data synthesis and reporting in systematic reviews and meta‐analyses on mechanistic studies?

'Step… drag… drag… step… step… wait… drag… step,' Frank Herbert writes in *Dune* (1965) to describe the so‐called sandwalk—a mystical, dance‐like but arrhythmic walk, seemingly changing direction abruptly, with a jittery and unpredictable style. This method, essential for non‐native travellers crossing the deadly deserts of the planet Arrakis, is used to avoid attracting the territorial and gigantic sandworms that dwell beneath the sands. Similarly, developing consensus guidelines for data synthesis and reporting in systematic reviews and meta‐analyses of mechanistic studies will inevitably be a ‘sandwalk’ between the different traditions and philosophies outlined above. But despite the thematic overlap, mechanistic research fundamentally differs from clinical research, focusing on causal claims regarding mechanisms rather than informing clinical decision‐making. This necessitates requirements that draw from, rather than merely copy, the best of EBM.

In our opinion, field‐specific guidelines for data synthesis and reporting in systematic reviews and meta‐analyses in mechanistic research would mandate a triangulation‐based approach that could potentially form the basis for field‐specific criteria to assess the certainty of evidence. While the exact implementation of triangulation remains to be determined, we propose the conceptual model presented in Figure 2. Put briefly, evidence supporting a mechanism is dependent findings that are replicable and documented by (1) multiple sources of data, that is, models, systems and/or settings; (2) multiple measurement methods; and (3) multiple experimental manipulations. Importantly, any conclusions about a mechanism should remain confined to the model, system and setting in which they were demonstrated. In essence, the more data sources, measurement methods and experimental manipulations that successfully document a mechanism, the greater the certainty of evidence. Ultimately, this approach could lead to the development of quantitative tools for evidence synthesis and a formal assessment of its certainty, akin to the GRADE (Grading of Recommendations, Assessment, Development and Evaluations) framework used in EBM (Neumann & Schünemann, 2024).

**FIGURE 2:**
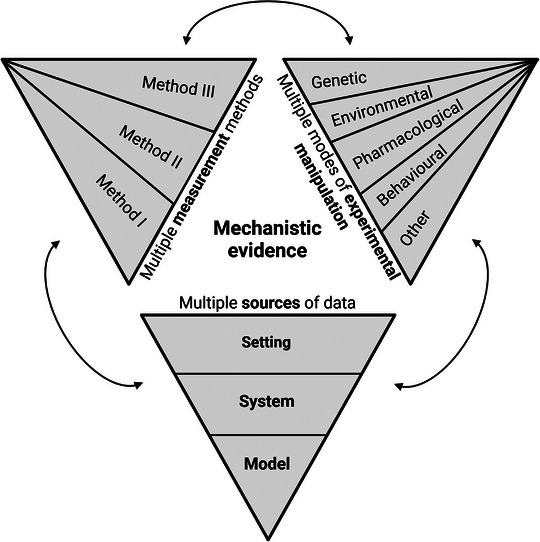
Proposed triangulation‐based concept to assess evidence in mechanistic research. Evidence supporting a mechanism depends on findings that across different studies, in addition to being replicable, are documented by: (1) multiple sources of data, that is models (in vitro, ex vitro, in vivo, other), systems (cell, tissue, isolated organ, organ system, organism) and settings (laboratory and applied); (2) multiple distinct measurement methods; and (3) multiple experimental manipulations. Any conclusions about a mechanism should remain confined to the model, system and setting in which they were demonstrated. The more data sources, measurement methods and experimental manipulations that successfully document a mechanism, the greater the certainty of the evidence. Created with BioRender.com.

As Frank Herbert wrote (Herbert, 1965): ‘He who controls the spice controls the universe.’ While evidence may be the ‘spice’ of any scientific inquiry, this is perhaps too daunting for any scientist when considering how difficult it is in reality to control even the simplest experiment, whether in the laboratory or applied setting. The systematic assessment of whether the results are confounded and whether they can be replicated and reproduced may help ensure that the ‘inductive leap’ to generalisability can be justified, enabling the integration of the mechanism into existing physiological theory. The spice must flow!

## AUTHOR CONTRIBUTIONS

Ronan M. G. Berg: conception, first draft, revisions. Cody G. Durrer: prepared figures, revisions. Jan Kyrre Berg Olsen Friis: revisions. Mathias Ried‐Larsen: conception, revisions. All authors have read and approved the final version of this manuscript and agree to be accountable for all aspects of the work in ensuring that questions related to the accuracy or integrity of any part of the work are appropriately investigated and resolved. All persons designated as authors qualify for authorship, and all those who qualify for authorship are listed.

## CONFLICT OF INTEREST

MR‐L is employed by Novo Nordisk A/S. Novo Nordisk A/S had no role in the decision to prepare, write, or publish the manuscript. No other authors have any conflict of interest to declare.

## FUNDING INFORMATION

The Centre for Physical Activity Research (CFAS) is supported by TrygFonden (grants ID 101390, ID 20045, ID 125132, and ID 177225). The funders had no role in study design, data collection and analysis, decision to publish, or preparation of the manuscript. C.G.D. was supported by the Canadian Institutes of Health Research (MFE‐176582). The funders had no role in the decision to prepare, write, or publish the manuscript.
